# Supplementary material for: Hyaluronic Acid-Modified and Doxorubicin-Loaded Gold Nanoparticles and Evaluation of Their Bioactivity
Source: Pharmaceuticals (Basel). 2021 Jan 28;14(2):101. doi: 10.3390/ph14020101 (PMC7911392; doi:10.3390/ph14020101)
Supplement: Supplementary file 1 [file pharmaceuticals-14-00101-s001.pdf]

Supporting Information for:

Hyaluronic acid-modified and doxorubicin-loaded gold nanoparticles and evaluation of their bioactivity

Bin Ren,[2,‡] Lin-Song Li,[1,‡] Xiaojing Yang,[1] Zhong-Chao Cai,[1] Xue-Jie Zhao[1] and Mei-Xia Zhao[1,*]

[1] Key Laboratory of Natural Medicine and Immuno-Engineering of Henan Province, Henan University, Jinming Campus, Kaifeng, Henan, 475004 (P.R. China)

[2] School of Mathematics and Statisticst, Henan University, Jinming Campus, Kaifeng, Henan, 475004 (P.R. China)

*Corresponding authors: E-mail:zhaomeixia2011@henu.edu.cn

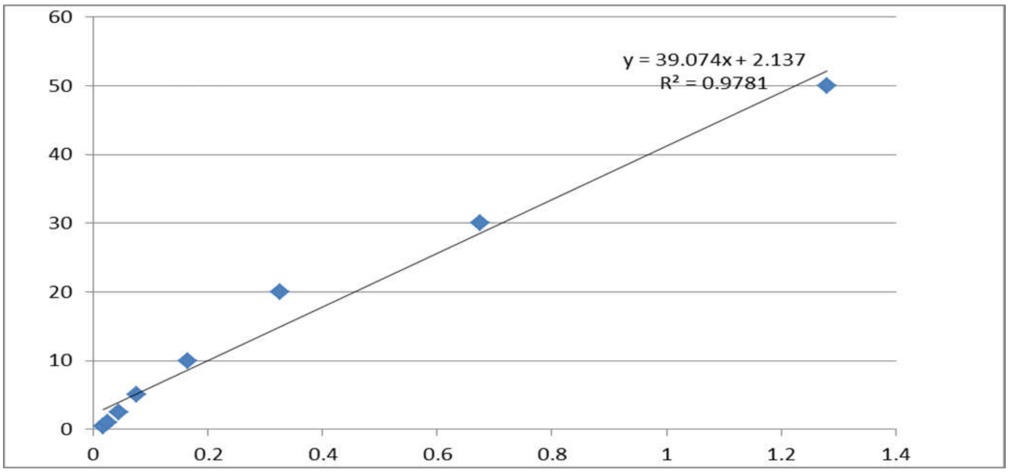

Figure S1. Standard UV-vis absorption curve of Au@MPAPEG-HA-ADH-Dox.

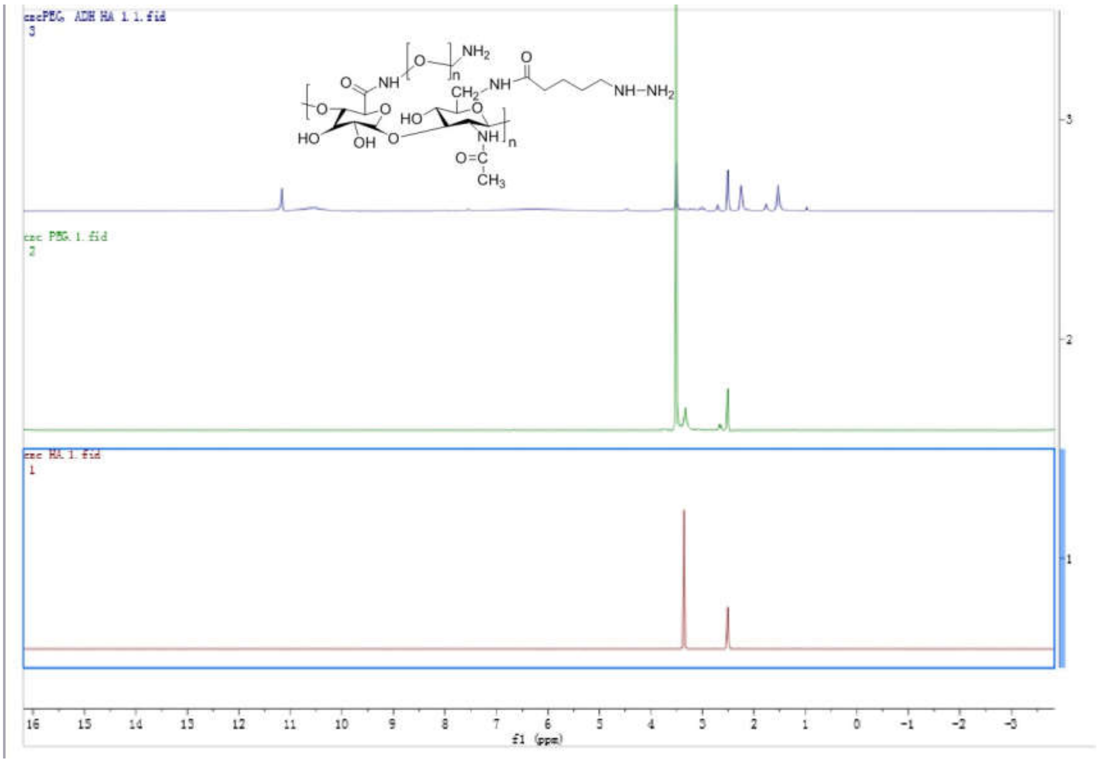

Figure S2. ^{1}H NMR of HA, PEG, and PEG-HA-ADH.

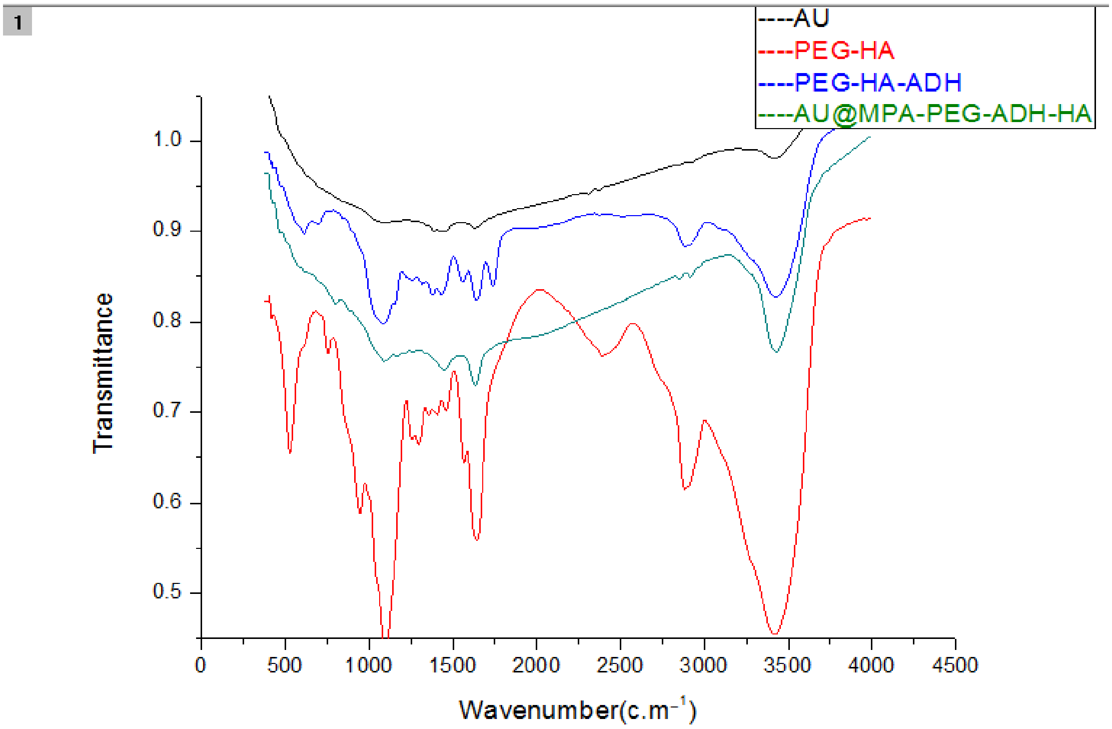

Figure S3. FTIR spectra of Au, PEG-HA, PEG-HA-ADH, and Au@MPA-PEG-HA-ADH.

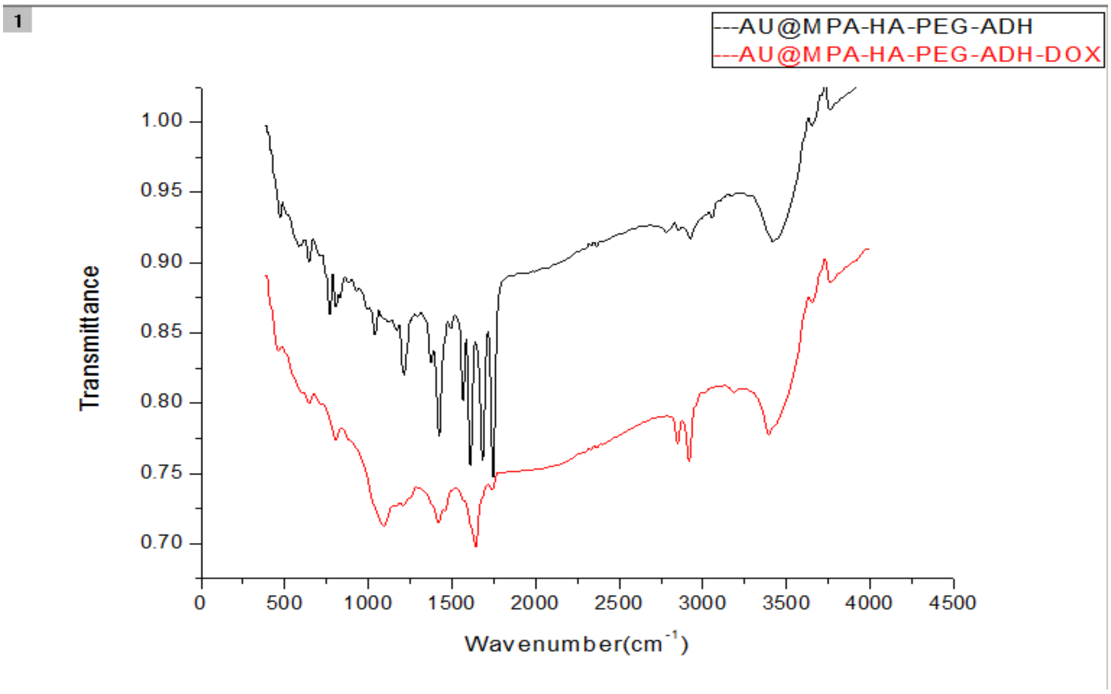

Figure S4. FTIR spectra of Au@MPA-PEG-HA-ADH and Au@MPA-PEG-HA-ADH-Dox.

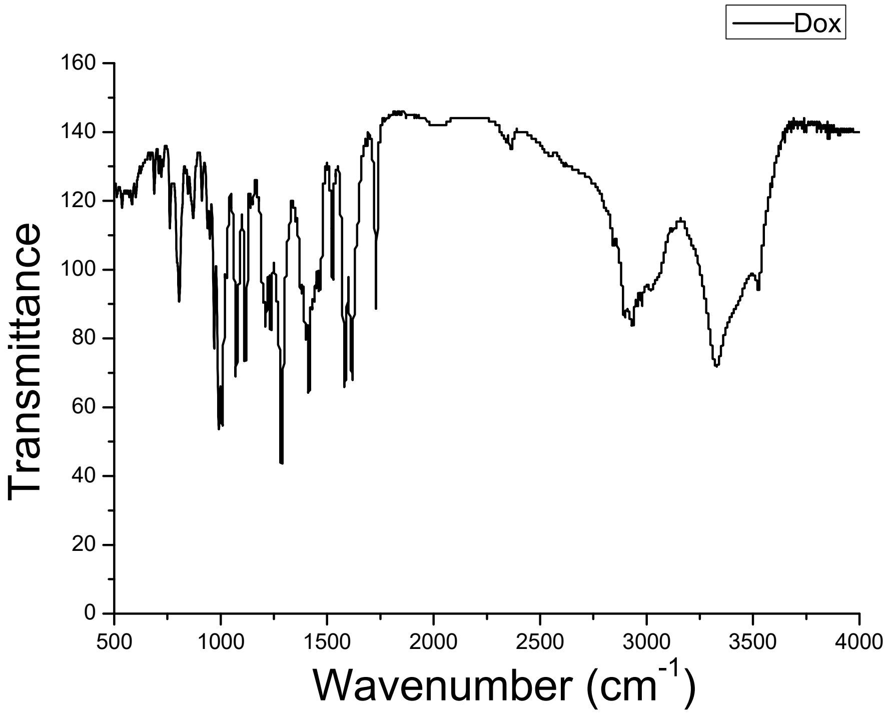

Figure S5. FTIR spectra of Dox.

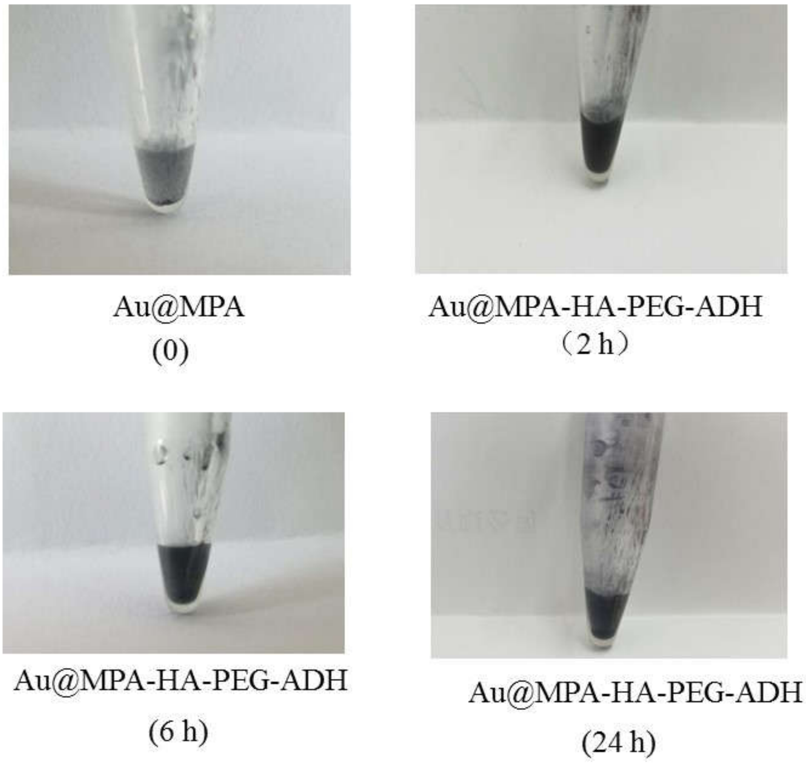

Figure S6. The variation of dispersion of nanoparticles at different resting times.

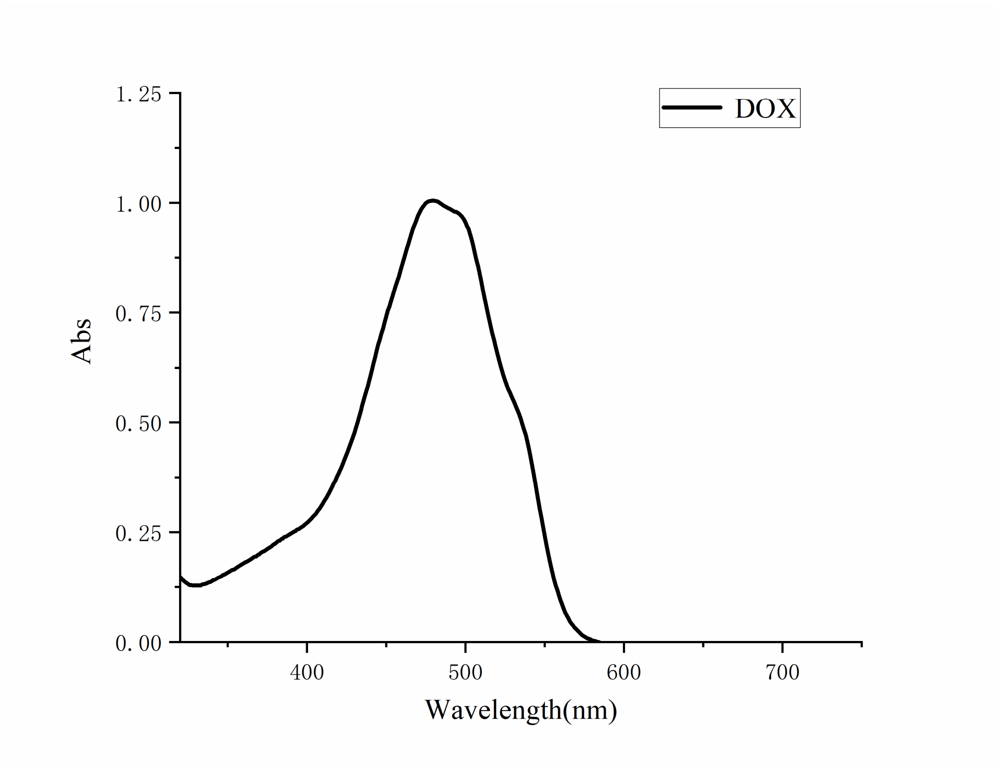

Figure S7. UV-vis spectra of Dox.
